# Induction and Maintenance Treatment of Lupus Nephritis: A Comprehensive Review of Meta-Analyses

**DOI:** 10.3390/jcm11020343

**Published:** 2022-01-11

**Authors:** Jae Il Shin, Han Li, Seoyeon Park, Jae Won Yang, Keum Hwa Lee, Yongsuk Jo, Seongeun Park, Jungmin Oh, Hansol Kim, Hyo Jin An, Gahee Jeong, Haerang Jung, Hyun Jung Lee, Jae Seok Kim, Seoung Wan Nam, Ai Koyanagi, Louis Jacob, Jimin Hwang, Dong Keon Yon, Seung-Won Lee, Kalthoum Tizaoui, Andreas Kronbichler, Ji Hong Kim, Lee Smith

**Affiliations:** 1 Department of Pediatrics, Yonsei University College of Medicine, Seoul 03722, Korea; shinji@yuhs.ac (J.I.S.); AZSAGM@yuhs.ac (K.H.L.); 2 University of Florida College of Medicine, Gainesville, FL 32610, USA; lih2@ufl.edu; 3 Yonsei University College of Medicine, Seoul 03722, Korea; harryme1713@yonsei.ac.kr (S.P.); ysjo1123@gmail.com (Y.J.); seongeun0413@yonsei.ac.kr (S.P.); jennyo333@yonsei.ac.kr (J.O.); fastdispel@hanmail.net (H.K.); pahj0622@naver.com (H.J.A.); 4 Department of Nephrology, Yonsei University Wonju College of Medicine, Wonju 26426, Korea; kidney74@yonsei.ac.kr (J.W.Y.); ripplesong@yonsei.ac.kr (J.S.K.); 5 Ewha Womans University College of Medicine, Seoul 07804, Korea; jgh1226s@naver.com (G.J.); gofkd2140@naver.com (H.J.); lhj_1215@hanmail.net (H.J.L.); 6 Department of Rheumatology, Yonsei University Wonju College of Medicine, Wonju 26426, Korea; dahsome@gmail.com; 7 Research and Development Unit, Parc Sanitari Sant Joan de Deu, Sant Boi de Llobregat, 08830 Barcelona, Spain; a.koyanagi@pssjd.org (A.K.); louis.jacob.contacts@gmail.com (L.J.); 8 Catalan Institution for Research and Advanced Studies, Pg. Lluis Companys 23, 08010 Barcelona, Spain; 9 Centro de Investigación Biomédica en Red de Salud Mental, 78019 Madrid, Spain; 10 Faculty of Medicine, University of Versailles Saint-Quentin-en-Yvelines, 78000 Montigny-le-Bretonneux, France; 11 Department of Epidemiology, Johns Hopkins Bloomberg School of Public Health, Baltimore, MD 21205, USA; jhwang56@jhu.edu; 12 Medical Science Research Institute, Kyung Hee University College of Medicine, Seoul 02447, Korea; yonkkang@gmail.com; 13 Department of Data Science, Sejong University College of Software Convergence, Sungkyunkwan University School of Medicine, Seoul 05006, Korea; lsw2920@gmail.com; 14 Laboratory of Microorganisms and Active Biomolecules, Sciences Faculty of Tunis, University Tunis El Manar, Tunis 1068, Tunisia; kalttizaoui@gmail.com; 15 Department of Medicine, University of Cambridge, Cambridge CB2 0QQ, UK; ak2283@cam.ac.uk; 16 Department of Pediatrics, Gangnam Severance Hospital, Yonsei University College of Medicine, Seoul 26426, Korea; 17 Centre for Health, Performance, and Wellbeing, Anglia Ruskin University, Cambridge CB1 1PT, UK; Lee.Smith@aru.ac.uk

**Keywords:** systemic lupus erythematosus, lupus nephritis, end-stage kidney disease, calcineurin inhibitor, glucocorticoids

## Abstract

Background: Lupus nephritis (LN) is present in over 50% of patients with systemic lupus erythematosus (SLE) which is managed with immunosuppressive and immunomodulatory therapies. However, several novel therapeutic approaches for LN are under investigation due to the adverse effects spectrum of conventional therapy; Methods: We performed a comprehensive review of meta-analyses aggregating the comparative efficacies of various pharmacotherapies for LN. We conducted a literature search and retrieved a total of 23 meta-analyses and network meta-analyses for summarization. Pharmacotherapies were evaluated across six major outcomes: remission, relapse, mortality, end stage kidney disease (ESKD) progression, infection, and malignancy. Result: Calcineurin inhibitors (CNI), particularly tacrolimus (TAC), in combination with glucocorticoids (GC) outperformed cyclophosphamide (CPA) with GC in the rate of remission, either complete or partial remission, and in terms of infectious complications. In maintenance therapy, MMF was superior to azathioprine (AZA) as the MMF-treated patients had lower relapse rate. Interpretation: This review aggregates evidence of therapy for clinicians and sheds light on comparative efficacies of alternative LN treatments. As more promising agents are entering the market, such as voclosporin, belimumab, and obinutuzumab, LN management might undergo significant changes during the next years.

## 1. Introduction

Systemic lupus erythematosus (SLE) is an autoimmune disease of unknown etiology, which can damage various tissues and organs [1]. Depending on the cohorts reported in literature 20–75% of SLE patients have lupus nephritis (LN) [2], and presence of LN is one of the major contributors to mortality in SLE patients [3]. Without adequate treatment, LN can lead to irreversible renal injury, end-stage kidney disease (ESKD), or death [4]. Treatment is in general subdivided into induction and maintenance therapy. Conventional treatment for LN induction therapy contained immunosuppressive drugs, specifically glucocorticoids (GC) combined with additional immunosuppressive measure such as cyclophosphamide (CPA), mycophenolate mofetil (MMF), and calcineurin inhibitors (CNI) including cyclosporine A (CSA) and tacrolimus (TAC) [5]. In maintenance, GC withdrawal is one aim of therapy and there is uncertainty to the duration of immunosuppressive therapy, which usually consists of MMF or azathioprine (AZA) or alternative management approaches. In addition to immunosuppression, immunomodulation with antimalarials as hydroxychloroquine or chloroquine are a mainstay of treatment [6]. Despite increased options of treatment, mortality and risk of ESKD have remained stable since the 1990s [7,8], underling the need for novel effective and safer treatment options.

Numerous clinical studies have compared efficacy and safety of these pharmacotherapies. However, there has not yet been a comprehensive review that compares all of these options. This review closes this knowledge gap highlighting treatments options for LN aiming to help clinicians understand current evidence as gathered by several meta-analyses.

## 2. Methods

### 2.1. Inclusion and Exclusion Criteria

Studies were included in our umbrella review if they were qualified for these following conditions: (1) estimated the efficacy of individual treatment for induction or maintenance of SLE using odds ratio (OR), relative risk (RR), risk difference (RD), or mean difference (MD), and 95% confidence interval (CI); (2) written in English; and (3) included a systematic review and meta-analysis. Articles were excluded if they met at least one of the following criteria: (1) irrelevant to pharmacotherapy for LN; (2) did not cover remission rates, relapse, mortality, progression to ESKD, and infection; (3) focused on Chinese or integrative medicine; (4) or non-human studies.

### 2.2. Search Strategy and Data Extraction

**Figure 1:**
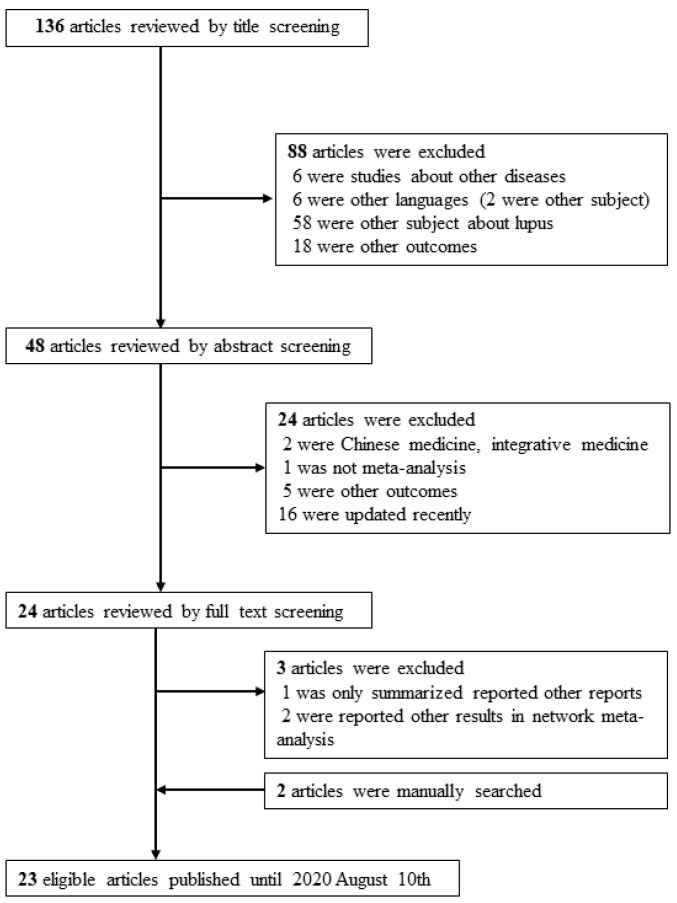
Flow chart of literature search.

Two authors performed a literature search in PubMed/Medline to find meta-analyses evaluating the efficacy of various induction or maintenance therapies for SLE from their inception to 30 August 2020, using the search term “lupus nephritis” AND “meta-analysis” (Figure 1). A manual search was also conducted to identify and include any relevant studies that could have been missed during the literature search.

Two investigators also documented outcome, comparison, first author, publication year, used models in meta-analysis (random or fixed effect models), type of metrics such as OR, RR, RD, or MD, summary effect with 95% CI, number of significant studies over total included studies, number of cases over controls, and reported *p*-value from each meta-analysis. Heterogeneity defined as I^2^ index (I^2^ > 50% is classified as high heterogeneity) [9], and Egger *p*-value (publication bias) were extracted from the articles if they were presented. Comparison of conventional meta-analysis and network meta-analysis was also recorded. Evaluation of statistical significancy was considered as two-tailed. We summarized reported summary of effects of meta-analysis compared to largest individual studies within each meta-analysis.

## 3. Results

Among 136 articles identified, 112 articles were excluded by title and abstract screening. The remaining 24 articles were further reviewed by full text and 3 of them were excluded because one only summarized a report and two reported other results in network meta-analysis. In addition, two articles were identified by a manual search. Therefore, 23 articles were eligible for this systemic review [4,5,10,11,12,13,14,15,16,17,18,19,20,21,22,23,24,25,26,27,28,29,30] (Figure 1).

### 3.1. Characteristics of Meta-Analysis

The 23 articles encompassed meta-analyses describing remission rates (complete and partial), relapse, mortality, ESKD progression, infection, and malignancy following different treatments for LN. None of the articles reported summary findings in an opposing direction to that of its largest study. Heterogeneity was fairly low: 8 studies reported I^2^ < 50%. Two studies used ORs as their primary metrics, and the remainder used RRs.

### 3.2. Complete and Partial Remission Rates during Induction Therapy in Lupus Nephritis

In comparison to CPA and GC, TAC in combination with GC demonstrated higher remission rates in both conventional and network meta-analyses (Table 1) [10]. CNI outperformed CPA in network meta-analyses [9]. In the meta-analyses for these respective findings, no individual study was significant, though a consistent effect was found when pooled (highest *p*-values: 0.20–0.23). There were no differences between Euro-Lupus regimen and NIH regimen of CPA [5]. MMF did not improve remission rates relative to CPA.

In two studies by Deng et al. and Tunnicliffe et al., a combination of MMF, TAC, and GC (“multitarget therapy”) increased complete remission rates over CPA and GC alone (RR 2.38; 95% CI: 1.07–5.30) [5,19]. Zhou et al., however, did not find differences in complete remission rates between these interventions (RR 5.13; 95% CI: 0.75–35.02), but in all three meta-analyses, the largest individual study effect size showed improved outcomes with multitarget therapy [5,11,19]. Furthermore, TAC improved complete remission rates when compared individually against other agents (RR 1.48; 95% CI: 1.23–1.77) and compositely with GC against CPA and GC (RR 2.41; 95% CI: 1.8–3.99) [10,20]. Regimens including two immunosuppressive agents (IS) improved complete remission rates over single IS regimens, though the effect size was small (RR 1.22; 95% CI: 1.09–1.35) [23]. Leflunomide with GC performed better than CPA and GC (RR 1.41; 95% CI: 1.10–1.82) [20]. Biologic agents did not improve complete remission rates, though rituximab in combination with MMF induced higher partial remission compared to MMF alone (RR 2.00; 95% CI: 1.05–3.82), a finding consistent with the largest study effect within that meta-analysis [5]. Additional findings are shown in Table 2. Network meta-analyses also showed higher partial remission rates for multitarget therapy compared to CPA and GC (RR 2.69; 95% CI: 1.74–4.76) (Table 3) [10].

### 3.3. Relapse during Maintenance Therapy in Lupus Nephritis

AZA did not affect relapse risk in comparison to CSA (RR 1.25; 95% CI: 0.51–3.06) or TAC (RR 6.62; 95% CI: 0.35–123.63) [5]. There was no difference between MMF + GC versus oral CPA + GC (RR 1.15; 95% CI: 0.55–2.37) or continuous GC versus GC withdrawal (RR 0.38; 95% CI: 0.05–2.88) [5]. However, in network meta-analyses, MMF reduced relapse risk over AZA (RR 0.53; 95% CI: 0.31–0.90) [10]. CPA was better than GC at reducing relapse (RR 0.31; 95% CI: 0.11–0.71) [4] (Table 4 and Table 5). In conventional meta-analyses, CPA and CNI demonstrated reduced relapse rates compared to other combinations.

### 3.4. Mortality during Induction Therapy in Lupus Nephritis

Only four conventional studies and one network study performed comparisons of mortality. Neither conventional nor network meta-analyses reported differences in mortality when MMF and CPA, CNI, AZA, or MMF alone were combined with GC as compared to CPA and GC alone. Oral CPA did not change mortality rates compared to intravenous (IV) CPA, and CPA combined with GC also did not change mortality relative to GC alone [5]. In the CPA + GC vs. GC and oral CPA vs. IV CPA comparisons, the reported summary effects, and network meta-analysis summary effects occurred in discordant directions (Table 6) [5,10]. Abatacept was the only biologic agent found to reduce mortality (RR 0.29; 95% CI: 0.10–0.91), an effect concordant with the largest individual study effect [5]. AZA with GC was also found to be more effective in mortality reduction than GC alone (RR 0.60; 95% CI: 0.36–0.99) (Table 7) [5].

### 3.5. Progression to ESKD during Induction Therapy in Lupus Nephritis

In the study by Tunnicliffe et al., CNI and GC did not change ESKD progression rates (Table 3) [5]. In network meta-analyses, CPA and GC outperformed GC alone in the reduction of ESKD incidence (total number of cases and controls: 385) (Table 8 and Table 9) [10]. Compared to AZA and MMF, high dose GC were associated with progression to ESKD and high CPA with GC and CPA with AZA were reducing ESKD incidence (Table 10) [16].

### 3.6. Infection during Induction Therapy in Lupus Nephritis

In conventional studies, CNI with GC had reduced infection risks than CPA and GC. Low CPA also had reduced infection rates relative to high CPA induction therapy [13]. No other differences regarding infection rates were shown in other comparisons, including MMF with GC and TAC with GC in either conventional or network meta-analyses (Table 11) [5,10,11,12]. Biologic-agents such as rituximab, abatacept, ocrelizumab, sirukumab, and belimumab did not affect infection incidence either alone or in conjunction with IS/CPA against placebo (Table 12) [5,27,29]. Network meta-analysis showed that GC with MMF or CNIs were associated with an increased incidence of infection compared to GC alone (Table 13) [25].

### 3.7. Relapse during Maintenance Therapy in Lupus Nephritis

No differences were found between CNI or CPA vs. AZA (Table 14) [13]. MMF was associated with reduced relapse rate as compared to AZA in network meta-analysis only, and this effect was concordant with the study with the largest effect size (Table 14) [16,18]. Heterogeneity was low in MMF vs. AZA and CNI vs. AZA comparisons.

### 3.8. Mortality during Maintenance Therapy in Lupus Nephritis

In conventional studies, no differences were found amongst MMF, AZA, or CPA as maintenance therapies in either summary findings or the largest effect within each meta-analysis [5,15]. Neither MMF, CNI, and GC combination therapy or plasma exchange with GC reduced mortality compared to CPA and GC in network meta-analyses (Table 15) [10].

### 3.9. Progression to ESKD during Maintenance Therapy in Lupus Nephritis

In conventional meta-analyses, MMF did not reduce ESKD progression compared to AZA, and AZA did not reduce ESKD progression relative to CPA [5,18]. In network meta-analyses, GC at normal and high doses resulted in higher ESKD progression rates than CPA + GC (RR 2.40; 95% CI: 1.05–5.48), CPA + AZA (RR 4.35; 95% CI: 1.56–14.28), MMF (RR 4.54; 95% CI: 1.45–17.31), and high-dose CPA + GC (RR 4.17; 95% CI: 1.41–16.67) [4].

### 3.10. Infection during Maintenance Therapy in Lupus Nephritis

AZA did not change infection rates versus MMF (RR 1.08; 95% CI: 0.60–1.96), though it was more effective than GC alone in conventional meta-analyses (RR 2.18; 95% CI: 1.01–4.73), as well as the largest study effect within that meta-analysis (Table 10) [5]. No differences were observed for CNI vs. AZA or GC (either withdrawal vs. continuation) [5,13]. In network meta-analyses, MMF (RR 5.50; 95% CI: 1.00–30.1) or CNI (RR 5.0; 95% CI: 1.1–22.6) with GC was more effective than GC alone to reduce infections (Table 15) [25]. However, there was no difference in infection rates between MMF + GC and CPA + GC across high and low CPA doses [14].

### 3.11. Malignancy during Induction and Maintenance Therapy in Lupus Nephritis

Based on conventional meta-analyses, no induction treatment regimen reduced incidence of malignancy [5]. In maintenance therapy, AZA was found to be equivalent to MMF (RR 4.04; 95% CI: 0.45–36.07) [5]. Network meta-analyses also found combined AZA and GC therapy to be equivalent to CPA and GC [10]. Varying CPA route of administration (oral versus IV), duration of therapy, or dosage did not affect malignancy risk [10] (Table 16 and Table 17).

## 4. Discussion

To address the need for a systematic comparison of available LN therapies, we collected and reviewed 23 meta-analyses evaluating comparative efficacies of LN drugs. To the best of our knowledge, we have conducted the most comprehensive review of meta-analyses of treatments use in the management of LN.

In induction therapy of LN, we found that CNI, particularly TAC, outperformed CPA in the rates of remission, alongside a beneficial safety profile. These effects were supported primarily by conventional meta-analyses, and improved outcome after TAC use in terms of remission was also supported by network meta-analyses. Multi-target therapy also improved remission rates, though without improvements in infectious risks. Defective regulatory T cells, increased activity of dendritic cells, and impaired clearance of neutrophil extracellular traps have been documented and thought to cause overactivation of T helper cells and B cells driving organ inflammation and injury in SLE [31,32,33]. CNI include TAC, which complexes FK506 binding protein 12, and CSA, which complexes with cyclophilin, and ultimately inhibit activity of the phosphatase calcineurin [26]. TAC and CSA have similar mechanisms of action, but TAC is approximately 25-times more potent than CSA and might thus be used primarily in the management of LN [26,34]. CNI prevent the calcineurin-mediated dephosphorylation and subsequent nuclear translocation of NF-AT, a transcription factor promoting IL-2 transcription. The use of CNI in real-life has been limited primarily due to the fact that clinical trials investigating either TAC or CSA have either been underpowered or of insufficient duration [31]. Our findings indicate that TAC with GCs outperform CPA with GCs across multiple moderate-sized meta-analyses. The beneficial effects of CNI have recently been confirmed in a phase 2 and phase 3 trial investigating the efficacy of voclosporin in combination with MMF [35,36]. Ethnicity plays a crucial role when assessing efficacy of agents in the management of LN, as CNI might be mainstay of therapy in Asian countries, while their efficacy might be comparable to conventional therapies in Caucasians [37,38,39].

Compared to IV cyclophosphamide, the use of calcineurin inhibitors (TAC and CSA) may be as effective in inducing complete renal remission, while the combination of MMF and TAC may improve the induction of complete renal remission, and achieving stable kidney function at six months. The generalizability of these findings may be limited as the two studies of combination therapy have largely included patients of Asian ethnicity, and have had serious concerns regarding selection bias and reporting bias. The safety of these therapies is unclear as the certainty of evidence is generally low to very low due to substantial imprecision in treatment effects and a small sample size and event numbers, limiting the applicability of the findings [5,37,40]. In Chinese patients CNIs have the potential to replace the more toxic CPA regimens in high-risk subgroups. TAC may be considered as another non- CPA alternative for induction therapy of LN and in those with refractory disease or intolerance to CPA or MMF.

Multitarget therapy of LN using TAC and MMF or GC versus TAC, MMF, and GC triple combination was analyzed for induction therapy. The combination TAC, MMF, and GC or TAC and GC provides better outcome data compared with CPA and GC. There is only good option for induction therapy of LN same evidence level.

For mortality, infection, progression to ESKD, malignancy, there is strong evidence for an impact of immunosuppressive agents. We can confirm that AZA with GC is reducing mortality rates when used as a maintenance agent, GC low dose reduces infection risk, and AZA with MMF reduces progression to ESKD in induction therapy of LN. These findings can explain that CNI or combination therapy are not suitable for maintenance therapy in LN and MMF or GC are good choices for remission maintenance in LN [4,5,10,13,16].

In the maintenance of remission, MMF outperformed AZA in reducing relapse, a finding which was supported by network but not conventional meta-analyses. Again, differences among ethnicities need to be taken into account. While in an international multicenter trial a maintenance strategy comparing MMF showed beneficial effects, these effects were not found in a European study [41,42]. MMF and AZA have distinct adverse effects and potential contraindications which are important to acknowledge when prescribing these agents. AZA may not be used in patients taking xanthine oxidase inhibitors or in patients with IBD, as AZA has a black box warning of hepatosplenic T-cell lymphoma in the context of IBD. MMF has a black box warning of embryo-fetal toxicity and thus should not be used in pregnant patients and patients who plan to conceive (men and women).

Antimalarial agents (HCQ) are inexpensive, especially compared with treatments used more recently in SLE patients, and also have a good overall tolerability profile, with the exception of a risk of retinal toxicity. To date, HCQ have not been reliably linked to an increased incidence of infections as has been known to occur with use of other immunosuppressive drugs [43]. Higher dosage use of HCQ can cause retinal toxicity, thus a dosage of 0.5-0.6 mg/kg/day maximum is suggested [44]. Despite reports of the valuable clinical effects of antimalarial agents in SLE, we still need additional confirmation from clinical studies as well as more-detailed basic studies to decipher their mechanisms of action [43].

Abatacept improved mortality over placebo when used as induction therapy. However, abatacept in combination with standard of care therapy failed to show higher remission rates in several clinical trials, thus it is neither used nor approved for treatment of LN [29,45]. Other biologic agents such as orcelizumab, sirukumab, laquinimod, and belimumab did not improve mortality, infection, and partial remission rates against placebo, either paired with immunosuppressants or alone in the case of belimumab. Rituximab and MMF had higher partial remission rates than MMF alone as induction therapy, but rituximab, either alone or combined with CPA, IS, or MMF, did not improve other outcomes. Abatacept, orcelizumab, and belimumab each had moderate-sized meta-analyses, but rituximab, laquinimod, and sirukumab had low sample sizes. Nonetheless, the BLISS-LN trial indicated that belimumab therapy has superior efficacy estimates and is thus approved for the management of LN. In this landmark trial, 448 patients underwent randomization to belimumab or placebo on top of standard therapy. The belimumab-treated cohort not only had higher complete response rates, but also reduced the risk of renal-related events or deaths [46].

Adverse effects should be considered to guide selection of therapy. Given the side effects of CPA and GCs, there is an impetus to evaluate CPA and GC at reduced intensities for induction therapy. Administration of lower cumulative doses of CPA or GC have been proposed to improve patient quality of life [47,48]. Modifying CPA or GC intensity had either no effect, or, in the case of low-dose CPA, detrimental effects on LN outcomes. The combination of newer pharmacologic agents not only allow the replacement of CPA but also indicates that a reduced GC regimen can be used safely. In a recent case series, belimumab allowed the withdrawal or reduction of GCs while maintaining a complete response [49]. Furthermore, the adverse effects of multitarget therapy consisting of a triple immunosuppressant combination, though effective at inducing remission, should be particularly considered given that adding pharmacotherapies may negatively impact quality of life. Multitarget therapy is most effective in class IV and class V LN lesions, and it has been reported that higher proportions of patients find the adverse effects of multitarget therapy intolerable compared to CPA and GC [19]. Multidrug therapy does not increase rates of infection or malignancy. For patients with more severe LN and more willing to tolerate adverse events, multidrug therapy may prove to be a superior option in inducing remission compared to double therapy.

Limitations are inherent to the design of the study. Sample size estimates of most interventions were low. Moreover, most interventions with low sample size were tested in one ethnicity and a generalizability of the results is not possible for some of these interventions, i.e., AZA is not an option to induce remission in Caucasians. Moreover, some trials such as the LUNAR trial investigating the role of rituximab on top of standard of care indicated higher overall remission rates [50]. As a negative trial, rituximab was not approved in the management of LN, but is still used in the management of LN, especially in refractory cases [51]. Moreover, analyses as exerted in this study always undergo a strict heterogeneity assessment. The highest heterogeneity in significant findings was I^2^ = 57% (multitarget therapy vs. CPA + GC), with the remainder of comparisons showing low heterogeneity (usually below I^2^ = 5%). Furthermore, we did not perform the assessment of the risk of bias and quality of evidence for network-meta-analyses. In network meta-analysis, the assessment of the risk of bias and quality of evidence are more challengeable than in conventional meta-analysis since network meta-analysis has more than one pooled effect estimate, which means that bias from a single trial could affect various pooled outcomes [52].

## 5. Conclusions

To conclude, we conducted a comprehensive review of meta-analysis on comparative efficacies for LN pharmacotherapy. Our study aggregates and interprets meta-analyses to guide clinicians and intensify discussion on optimal LN care. We find that TAC and GC or multitarget therapy are particularly effective at inducing remission without increases in infection or malignancy. For maintenance of LN after remission, MMF and GC are reducing mortality, infection, progression to ESKD, and relapse. Preliminary evidence is promising for biologics, but further evidence is required to demonstrate superior mortality and quality of life outcomes. Research in SLE and LN is a rapidly moving field and novel therapies will change the landscape in near future.

## Figures and Tables

**Table 1:** Remission rates after induction therapy in lupus nephritis (comparison of the results between conventional meta-analysis and network meta-analysis).

Comparison	Author, Year	M	T	Reported Summary Effect (95% CI)	NMA Reported Summary Effect (95% CI)	Reported *p* Value	I2 (*p* Value)	No. of Significant Study/Total Study	No. of Cases/Controls	Largest Effect (95% CI)	*p* Value (Largest)	NMA Author, Year
MMF + GC vs. CPA + GC	Tunnicliffe, et al., 2018 [5]	R	RR	1.17 (0.97–1.42)	1.44 (1.00–2.06)	0.11	0% (0.46)	1/10	868/441	1.07 (0.54–2.09)	-	Palmer, et al., 2017 [10]
MMF + CPA + GC vs. CPA + GC	Tunnicliffe, et al., 2018 [5]	F	RR	1.22 (0.78–1.89)	1.48 (0.62–3.53)	0.38	-	0//1	82/40	1.22 (0.78–1.89)	0.38	Palmer, et al., 2017 [10]
TAC + GC vs. CPA + GC	Zhou, et al., 2019 [11]	F	RR	2.41 (1.8–3.99)	2.35 (1.03–5.45)	0.0006	0% (0.93)	0/7	276/135	1.64 (0.65–4.14)	0.20	Lee, et al., 2015 [12]
CNI + GC vs. CPA + GC	Zhang, et al., 2016 [13]	F	RR	1.33 (0.93–1.90)	1.74 (1.09–2.79)	0.12	0% (0.97)	0/4	188/95	1.36 (0.83–2.22)	0.20	Palmer, et al., 2017 [10]
CPA + GC vs. GC	Tunnicliffe, et al., 2018 [5]	F	RR	2.63 (0.13–54.64)	0.57 (0.23–1.40) *	0.53	-	0/1	13/7	2.63 (0.13–54.64)	0.53	Palmer, et al., 2017 [10]
AZA + GC vs. CPA + GC	Tunnicliffe, et al., 2018 [5]	F	RR	2.03 (0.64–6.46)	1.53 (1.53–2.39) †	0.23	-	0/1	59/38	2.03 (0.64–6.46)	0.23	Singh, et al., 2016 [4]
Low CPA vs. High CPA	Tunnicliffe, et al., 2018 [5]	R	RR	1.09 (0.63–1.86)	1.46 (0.83–2.86)	0.76	67% (0.05)	0/3	267/117	1.37 (0.75–2.50)	0.29	Bae, et al., 2019 [14]
												
TAC + GC vs. MMF + GC	Zhou, et al., 2019 [11]	F	RR	0.95 (0.54–1.64)	1.60 (0.70–3.57)	0.84	42% (0.18)	0/3	206/103	1.13 (0.59–2.18)	0.71	Lee, et al., 2015 [12]

Abbreviations: CI: confidence interval; CNI: calcineurin inhibitors; CPA: cyclophosphamide; F: fixed effect; GC: glucocorticoid; Largest effect: effect of the largest individual study in the meta-analysis; M: model; No: Number; NMA: Network meta-analysis; MMF: Mycophenolate mofetil; R: random effect; RR: relative risk; T: type of metrics; TAC: tacrolimus. * results of GC vs. CPA + GC; † results of CPA + GC vs. AZA + GC; results of overall effect.

**Table 2:** Complete and partial remission rates of induction therapy in lupus nephritis.

Comparison	Author, Year	M	Type of Metrics	Reported Summary Effect (95% CI)	Reported *p* Value	I2 (*p* Value)	No. of Significant Study/Total Study	No. of Cases/Controls	Largest Effect (95% CI)	*p* Value (Largest)
Complete Remission										
CPA vs. Others										
MMF + TAC + GC vs. CPA + GC	Tunnicliffe, et al., 2018 [5]	R	RR	2.38 (1.07–5.30)	0.034	57% (0.13)	2/2	402/201	1.80 (1.34–2.42)	<0.001
MMF + TAC + GC vs. CPA + GC	Zhou, et al., 2019 [11]	R	RR	5.13 (0.75–35.02)	0.10	69% (0.07)	1/2	402/201	2.49 (1.59–3.88)	<0.001
MMF + GC vs. Oral CPA + GC	Tunnicliffe, et al., 2018 [5]	F	RR	0.98 (0.74–1.30)	0.88	-	0/1	62/30	0.98 (0.74–1.30)	0.88
Multi target vs. CPA	Deng, et al., 2018 [18]	F	RR	1.94 (1.61–2.33)	<0.001	0% (0.48)	6/8	801/406	1.80 (1.34–2.42)	<0.001
Leflunomide +GC vs. CPA + GC	Cao, et al., 2015 [20]	F	RR	1.41 (1.10–1.82)	0.007	0% (0.94)	1/9	442/220	1.63 (1.03–2.56)	-
AZA + CPA+ GC vs. GC	Tunnicliffe, et al., 2018 [5]	F	RR	0.21 (0.04–1.02)	0.054	-	0/1	29/7	0.21 (0.04–1.02)	-
CIs vs. Others										
MMF + CPA + GC vs. CPA + GC	Tunnicliffe, et al., 2018 [5]	F	RR	1.22 (0.78–1.89)	0.38	-	0//1	82/40	1.22 (0.78–1.89)	0.38
TAC vs. Others	Karaaij, et al., 2016 [21]	F	RR	1.48 (1.23–1.77)	<0.05	65% (0.021)	2/5	693/-	1.77 (1.31–2.38)	<0.001
MMF + GC vs. TAC + GC	Tunnicliffe, et al., 2018 [5]	R	RR	1.02 (0.83–1.26)	0.85	0% (0.59)	0/3	273/135	0.95 (0.74–1.23)	0.71
TAC + GC vs. CPA + GC	Zhou, et al., 2019 [11]	F	RR	2.41 (1.46–3.99)	0.0006	0% (0.93)	0/7	276/135	1.64 (0.65–4.14)	0.20
Biologic agents										
Rituximab vs. Others	Zhong, et al., 2019 [22]	R	RR	1.98 (0.90–4.39)	0.09	68% (0.02)	1/5	548/378	1.40 (0.53–3.75)	0.16
Rituximab + CPA vs. Rituximab	Tunnicliffe, et al., 2018 [5]	F	RR	0.90 (0.16–5.13)	0.91	-	0/1	19/9	0.90 (0.16–5.13)	0.91
Rituximab + MMF vs. MMF	Tunnicliffe, et al., 2018 [5]	F	RR	0.86 (0.51–1.45)	0.58	-	0/1	144/72	0.86 (0.51–1.45)	0.58
Abatacept + IS vs. placebo + IS	Tunnicliffe, et al., 2018 [5]	R	RR	1.13 (0.74–1.71)	0.57	0% (0.74)	0/2	432/168	1.08 (0.66–1.77)	-
Laquinimod + IS vs. placebo + IS	Tunnicliffe, et al., 2018 [5]	F	RR	1.55 (0.73–3.42)	0.28	-	0/1	46/15	1.55 (0.70–3.42)	0.28
Orcrelizumab + IS vs. placebo + IS	Tunnicliffe, et al., 2018 [5]	F	RR	1.07 (0.74–1.56)	0.72	-	0/1	223/75	1.07 (0.74–1.56)	0.72
Sirukumab + IS vs. placebo + IS	Tunnicliffe, et al., 2018 [5]	F	RR	0.93 (0.66–1.32)	0.70	-	0/1	25/4	0.93 (0.66–1.32)	0.70
Others										
GC vs. reduced GC	Tunnicliffe, et al., 2018 [5]	F	RR	0.93 (0.39–2.23)	0.87	-	0/1	81/39	0.93 (0.39–2.23)	0.87
GC + 2 IS vs. GC + 1 IS	Liu, et al., 2019 [23]		RR	1.22 (1.09–1.35)	<0.001	30.5% (0.17)	3/10	1432/622	1.32 (1.16–1.51)	<0.001
AZA + GC vs. GC	Tunnicliffe, et al., 2018 [5]	R	RR	0.95 (0.54–1.69)	0.87	2% (0.31)	0/2	37/19	0.91 (0.56–1.46)	-
Partial Remission										
CPA vs. Others										
MMF + TAC + GC vs. CPA + GC	Tunnicliffe, et al., 2018 [5]	R	RR	1.00 (0.78–1.28)	1.00	0% (1.00)	0/2	402/201	1.00 (0.77–1.30)	-
AZA + GC vs. CPA + GC	Tunnicliffe, et al., 2018 [5]	R	RR	1.80 (0.67–4.81)	0.24	-	0/1	59/38	1.80 (0.67–1.81)	0.24
MMF + GC vs. CPA + GC	Tunnicliffe, et al., 2018 [5]	R	RR	1.02 (0.89–1.18)	0.74	0% (0.96)	0/9	868/441	1.06 (0.85–1.32)	-
TAC + GC vs. CPA + GC	Deng, et al., 2012 [24]	F	RR	0.95 (0.68–1.33)	0.78	0% (0.63)	0/5	219/111	0.87 (0.52–1.48)	0.20
CNI + GC vs. CPA + GC	Tunnicliffe, et al., 2018 [5]	R	RR	0.86 (0.61–1.26)	0.48	0% (0.76)	0/4	178/87	0.82 (0.50–1.36)	-
Low vs. High CPA	Tian, et al., 2017 [17]	F	RR	1.08 (0.89–1.32)	0.43	0% (0.92)	0/3	411/220	1.12 (0.85–1.48)	0.41
MMF + GC vs. oral CPA + GC	Tunnicliffe, et al., 2018 [5]	F	RR	1.07 (0.44–2.59)	0.88	-	0/1	62/30	1.07 (0.44–2.59_	0.88
Others										
MMF + CPA + GC vs. CPA + GC	Tunnicliffe, et al., 2018 [5]	F	RR	1.03 (0.55–1.90)	0.94	-	0/1	82/40	1.03 (0.55–1.90)	0.94
MMF + GC vs. TAC + GC	Tunnicliffe, et al., 2018 [5]	R	RR	0.83 (0.51–1.36)	0.47	0% (0.66)	0/2	190/94	0.78 (0.44–1.33)	-
MMF + Rituximab vs. MMF	Tunnicliffe, et al., 2018 [5]	F	RR	2.00 (1.05–3.82)	0.035	-	1/1	144/72	2.00 (1.05–3.82)	0.035
Rituximab + CPA vs. Rituximab	Tunnicliffe, et al., 2018 [5]	F	RR	0.75 (0.35–1.62)	0.47	-	0/1	19/9	0.75 (0.35–1.62)	0.47
Abatacept + IS vs. placebo + IS	Tunnicliffe, et al., 2018 [5]	R	RR	0.88 (0.58–1.33)	0.54	0% (0.80)	0/2	432/168	0.92 (0.53–1.61)	-
Orcrelizumab + IS vs. placebo + IS	Tunnicliffe, et al., 2018 [5]	F	RR	1.49 (0.82–2.49)	0.13	-	0/1	223/75	1.49 (0.89–2.49)	0.13
GC vs. reduced GC	Tunnicliffe, et al., 2018 [5]	F	RR	1.33 (0.78–2.24)	0.29	-	0/1	81/39	1.33 (0.78–2.24)	0.29

Abbreviations: AZA: azathioprine; CI: confidence interval; CNI: calcineurin inhibitors; CPA: cyclophosphamide; F: fixed effect; GC: glucocorticoid; IS: immunosuppressive agents; Largest effect: effect of the largest individual study in the meta-analysis; M: model; No: Number; NMA: Network meta-analysis; MMF: Mycophenolate mofetil; OR: odds ratios; R: random effect; RR: relative risk; TAC: tacrolimus.

**Table 3:** Complete and partial remission rates of induction therapy in lupus nephritis (Network meta-analysis).

Comparison	Author, Year	Reported Summary Effect (95% CI)
CPA vs. Others		
MMF + GC vs. Low CPA + GC	Bae, et al., 2019 [14]	1.02 (0.51–2.02) *
MMF + GC vs. High CPA + GC	Bae, et al., 2019 [14]	1.48 (0.99–2.44) *
MMF + CIs + GC vs. CPA + GC	Palmer, et al., 2017 [10]	2.69 (1.74–4.76)
Low CPA + GC vs. CSA + GC	Singh, et al., 2016 [4]	0.38 (0.38–0.76) *,†
High CPA + GC vs. CSA + GC	Singh, et al., 2016 [4]	0.55 (0.55–0.87) *,†
Low CPA vs. TAC	Singh, et al., 2016 [4]	0.50 (0.50–0.95) *
Low CPA vs. CPA	Singh, et al., 2016 [4]	0.51 (0.51–0.95)
Oral CPA vs. CPA	Palmer, et al., 2017 [10]	0.57 (0.23–1.40)
Mizoribine + GC vs. CPA + GC	Palmer, et al., 2017 [10]	0.29 (0.08–1.11)
Other		
CNI + GC vs. MMF + GC	Tang, et al., 2018 [25]	1.0 (0.3–3.6)
Plasma exchange vs. CSA	Singh, et al., 2016 [4]	0.49 (0.49–0.97) *
MMF +GC vs. GC	Tang, et al., 2018 [25]	8.6 (1.4–51.6)
CNI + GC vs. GC	Tang, et al., 2018 [25]	8.8 (1.6–48.2)

Abbreviations: CNI: calcineurin inhibitors; CPA: cyclophosphamide; GC: glucocorticoid; MMF: mycophenolate mofetil; TAC: tacrolimus. * reports of overall effects; † includes partial remission, complete remission, and renal response.

**Table 4:** Relapse during maintenance therapy of lupus nephritis.

Comparison	Author, Year	M	Type of Metrics	Reported Summary Effect (95% CI)	Reported *p* Value	I2 (*p* Value)	No. of Significant Study/Total Study	No. of Cases/Controls	Largest Effect (95% CI)	*p* Value (Largest)
AZA vs. CSA	Tunnicliffe, et al., 2018 [5]	F	RR	1.25 (0.51–3.06)	0.63	-	0/1	69/36	1.25 (0.51–3.06)	0.63
AZA vs. TAC	Tunnicliffe, et al., 2018 [5]	F	RR	6.62 (0.35–123.63)	0.21	-	0/1	70/34	6.62 (0.35–123.63)	0.21
MMF + GC vs. oral CPA + GC	Tunnicliffe, et al., 2018 [5]	F	RR	1.15 (0.55–2.37)	0.71	-	0/1	62/30	1.15 (0.55–2.37)	0.71
GC withdrawal vs. GC continuous	Tunnicliffe, et al., 2018 [5]	R	RR	0.38 (0.05–2.88)	0.35	-	0/1	15/8	0.38 (0.05–2.88)	0.35

Abbreviations: AZA: azathioprine; CI: confidence interval; CPA: cyclophosphamide; CSA: cyclosporine A; F: fixed effect; GC: glucocorticoid; Largest effect: effect of the largest individual study in the meta-analysis; M: model; No: Number; MMF: Mycophenolate mofetil; OR: odds ratios; R: random effect; RR: relative risk.

**Table 5:** Relapse during maintenance therapy of lupus nephritis (Network meta-analysis).

Comparison	Author, Year	Reported Summary Effect (95% CI)
MMF vs. AZA	Palmer, et al., 2017 [10]	0.53 (0.31–0.90)
CPA vs. GC	Singh, et al., 2016 [4]	0.31 (0.11–0.71)

Abbreviations: AZA: azathioprine; CPA: cyclophosphamide; GC: glucocorticoid; MMF: mycophenolate mofetil.

**Table 6:** Mortality during induction therapy (compared with Network meta-analysis).

Comparison	Author, Year	M	Ta	Reported Summary Effect (95% CI)	NMA Reported Summary Effect (95% CI)	Reported *p* Value	I2 (*p* Value)	No. of Significant Study/Total Study	No. of Cases/Controls	Largest Effect (95% CI)	*p* Value (Largest)	NMA Author, Year
MMF + CPA + GC vs. CPA + GC	Tunnicliffe, et al., 2018 [5]	F	RR	0.95 (0.06–14.72)	0.92 (0.06–15.3)	0.97	-	0//1	82/40	0.96 (0.06–14.72)	0.97	Palmer, et al., 2017 [10]
CNI + GC vs. CPA + GC	Tunnicliffe, et al., 2018 [5]	R	RR	0.41 (0.06–2.69)	0.83 (0.27–2.56)	0.36	0% (0.79)	0/3	153/75	0.29 (0.01–6.93)	0.50	Palmer, et al., 2017 [10]
CPA + GC vs. GC	Tunnicliffe, et al., 2018 [5]	R	RR	0.98 (0.53–1.82)	2.03 (0.72–5.77) *	0.94	10% (0.35)	0/4	226/88	4.91 (0.66–36.40)	-	Palmer, et al., 2017 [10]
AZA + GC vs. CPA + GC	Tunnicliffe, et al., 2018 [5]	R	RR	1.39 (0.25–7.77)	1.52 (0.52–4.46)	0.71	67% (0.08)	0/2	146/75	0.49 (0.09–2.81)	-	Palmer, et al., 2017 [10]
MMF + GC vs. CPA + GC	Henderson, et al., 2013 [15]	R	RR	1.02 (0.52–1.98)	4.20 (0.59–2.44)	0.96	0% (0.54)	0/4	710/349	1.02 (0.61–5.27)	0.24	Palmer, et al., 2017 [10]
Oral CPA vs. IV CPA	Tunnicliffe, et al., 2018 [5]	R	RR	0.80 (0.20–3.24)	2.86 (0.82–10.0)	0.76	34% (0.22)	0/2	67/34	0.51 (0.18–1.47)	-	Palmer, et al., 2017 [10]

Abbreviations: AZA: azathioprine; CI: confidence interval; CNI: calcineurin inhibitors; CPA: cyclophosphamide; F: fixed effect; GC: glucocorticoid; Largest effect: effect of the largest individual study in the meta-analysis; M: model; No: Number; NMA: Network meta-analysis; MMF: Mycophenolate mofetil; R: random effect; RR: relative risk. * results of GC vs. CPA + GC; aType of metrics.

**Table 7:** Mortality during induction and maintenance therapy in lupus nephritis.

Comparison	Author, Year	M	Type of Metrics	Reported Summary Effect (95% CI)	Reported *p* Value	I2 (*p* Value)	No. of Significant Study/Total Study	No. of Cases/Controls	Largest Effect (95% CI)	*p* Value (Largest)
Induction therapy										
CPA vs. Others										
Low CPA vs. High CPA	Tunnicliffe, et al., 2018 [5]	R	RR	0.97 (0.14–6.56)	0.98	-	0/2	121/63	0.97 (0.14–6.56)	-
CPA + AZA + GC vs. GC	Tunnicliffe, et al., 2018 [5]	R	RR	0.53 (0.17–1.68)	0.28	-	0/1	29/7	0.53 (0.17–1.68)	0.28
CNI vs. Others										
TAC vs. Others	J. Hannah, et al., 2016 [25]	F	RR	0.39 (0.10–1.42)	0.15	0% (1.00)	0/5	153/75	0.29 (0.01–6.93)	0.5
MMF + GC vs. TAC + GC	Tunnicliffe, et al., 2018 [5]	R	RR	1.10 (0.44–2.77)	0.84	0% (0.98)	0/7	273/135	1.17 (0.37–3.66)	1.00
Biologic agents										
Rituximab + MMF vs. MMF	Tunnicliffe, et al., 2018 [5]	F	RR	5.00 (0.24–102.35)	0.30	-	0/1	144/72	5.00 (0.24–102.35)	0.30
Belimumab vs. placebo	Kandala, et al., 2013 [27]	R	RR	0.59 (0.16–2.11)	-	-	0/3	2133/675	-	-
Abatacept + IS vs. placebo + IS	Tunnicliffe, et al., 2018 [5]	R	RR	0.29 (0.10–0.91)	0.034	0% (0.92)	1/2	432/168	0.29 (0.09–0.96)	-
Laquinimod + IS vs. placebo + IS	Tunnicliffe, et al., 2018 [5]	F	RR	1.50 (0.06–34.79)	0.80	-	0/1	46/15	1.50 (0.06–34.79)	0.80
Orcrelizumab + IS vs. placebo + IS	Tunnicliffe, et al., 2018 [5]	F	RR	0.66 (0.23–1.85)	0.43	-	0/1	379/125	0.66 (0.23–1.85)	0.43
Others										
GC vs. reduced GC	Tunnicliffe, et al., 2018 [5]	F	RR	4.65 (0.23–93.95)	0.32	-	0/1	81/39	4.65 (0.23–93.95)	0.32
Plasma exchange + IS vs. IS	Tunnicliffe, et al., 2018 [5]	R	RR	1.62 (0.64–4.09)	0.31	-	0/2	125/65	1.53 (0.58–4.04)	-
AZA + GC vs. GC	Tunnicliffe, et al., 2018 [5]	R	RR	0.60 (0.36–0.99)	0.048	0% (0.74)	1/3	78/35	0.53 (0.29–0.99)	-
Maintenance therapy										
MMF vs. AZA	Deng, et al., 2019 [18]	F	RR	0.55 (0.23–1.28)	0.16	0% (0.58)	0/7	601/294	0.32 (0.01–7.82)	-
AZA vs. CPA	Tunnicliffe, et al., 2018 [5]	R	RR	0.12 (0.01–2.03)	0.14	-	0/1	39/20	0.12 (0.01–2.03)	0.14

Abbreviations: AZA: azathioprine; CI: confidence interval; CNI: calcineurin inhibitors; CPA: cyclophosphamide; F: fixed effect; GC: glucocorticoid; IS: immunosuppressive agents; Largest effect: effect of the largest individual study in the meta-analysis; M: model; No: Number; MMF: Mycophenolate mofetil; OR: odds ratios; R: random effect; RR: relative risk; TAC: tacrolimus.

**Table 8:** Progression to ESRD (compared with Network meta-analysis).

Comparison	Author, Year	M	T	Reported Summary Effect (95% CI)	NMA Author, Year	NMA Reported Summary Effect (95% CI)	Reported *p* Value	I2 (*p* Value)	No. of Significant Study/Total Study	No. of Cases/Controls	Largest Effect (95% CI)	*p* Value (Largest)
MMF + GC vs. CPA + GC	Tunnicliffe, et al., 2018 [5]	R	RR	0.71 (0.27–1.84)	Palmer, et al., 2017 [10]	2.60 (0.36–18.7)	0.48	0% (0.65)	0/3	231/117	0.53 (0.15–1.81)	0.31
CNI + GC vs. CPA + GC	Tunnicliffe, et al., 2018 [5]	F	RR	1.00 (0.07–14.85)	Palmer, et al., 2017 [10]	2.08 (0.23–18.9)	1.0	-	0/1	38/19	1.00 (0.07–14.85)	-
CPA + GC vs. GC	Tunnicliffe, et al., 2018 [5]	R	RR	0.63 (0.39–1.03)	Singh, et al., 2016 [16]	0.56 (0.33–0.92)	0.07	0% (0.87)	0/4	278/107	0.74 (0.29–1.86)	-
AZA + GC vs. CPA + GC	Tunnicliffe, et al., 2018 [5]	R	RR	0.40 (0.15–1.07)	Palmer, et al., 2017 [10]	1.79 (0.56–5.70)	0.07	0% (0.76)	0/2	144/56	0.25 (0.01–5.93)	-
Oral CPA vs. IV CPA	Tunnicliffe, et al., 2018 [5]	R	RR	0.23 (0.04–1.28)	Palmer, et al., 2017 [10]	1.34 (0.31–5.88)	0.09	0% (0.97)	0/2	67/34	0.23 (0.03–1.83)	-

Abbreviations: AZA: azathioprine; CI: confidence interval; CNI: calcineurin inhibitors; CPA: cyclophosphamide; F: fixed effect; GC: glucocorticoid; Largest effect: effect of the largest individual study in the meta-analysis; M: model; No: Number; OR: odds ratios; R: random effect; RR: relative risk; T: type of metrics.

**Table 9:** Progression to ESRD during treatment of lupus nephritis.

Comparison	Author, Year	M	Type of mETRICS	Reported Summary Effect (95% CI)	Reported *p* Value	I2 (*p* Value)	No. of Significant Study/Total Study	No. of Cases/Controls	Largest Effect (95% CI)	*p* Value (Largest)
Induction therapy										
CPA vs. Others										
MMF + GC vs. oral CPA + GC	Tunnicliffe, et al., 2018 [5]	F	RR	0.19 (0.01–3.76)	0.27	-	0/1	62/30	0.19 (0.01–3.76)	0.27
CPA + AZA + GC vs. GC	Tunnicliffe, et al., 2018 [5]	F	RR	0.21 (0.04–1.02)	0.054	-	0/1	29/7	0.21 (0.04–1.02)	0.054
Low vs. High CPA	Tunnicliffe, et al., 2018 [5]	R	RR	0.49 (0.05–5.20)	0.55	-	0/2	135/64	0.49 (0.05–5.20)	0.55
Long vs. Short CPA	Tunnicliffe, et al., 2018 [5]	R	RR	0.40 (0.09–1.83)	0.24	-	0/1	40/20	0.40 (0.09–1.83)	0.24
CNs vs. Others										
MMF + GC vs. TAC + GC	Tunnicliffe, et al., 2018 [5]	R	RR	1.22 (0.51–2.91)	0.66	-	0/1	150/74	1.22 (0.51–2.91)	0.66
Biologic agents										
Abatacept + IS vs. placebo + IS	Tunnicliffe, et al., 2018 [5]	R	RR	0.84 (0.21–3.45)	0.81	-	0/1	298/100	0.84 (0.21–3.45)	0.81
Others										
GC vs. reduced GC	Tunnicliffe, et al., 2018 [5]	F	RR	0.93 (0.39–2.23)	0.87	-	0/1	81/39	0.93 (0.39–2.23)	0.87
AZA + GC vs. GC	Flanc, et al., 2004 [28]	R	RR	0.66 (0.17–2.55)	0.55	49.7% (0.16)	0/2	54/22	0.33 (0.08–1.32)	-
Plasma exchange + IS vs. IS	Tunnicliffe, et al., 2018 [5]	R	RR	1.24 (0.60–2.57)	0.56	0% (0.63)	0/3	143/74	1.44 (0.63–3.29)	-
Plasma exchange vs. IS	Tunnicliffe, et al., 2018 [5]	F	RR	0.24 (0.01–4.44)	0.34	-	0/1	20/11	0.24 (0.01–4.44)	0.34
Maintenance therapy										
MMF vs. AZA	Deng, et al., 2019 [18]	F	RR	0.50 (0.18–1.39)	0.19	0% (0.74)	0/5	514/252	0.14 (0.01–2.62)	0.07
AZA vs. CPA	Tunnicliffe, et al., 2018 [5]	R	RR	0.35 (0.04–3.09)	0.35	-	0/1	39/20	0.35 (0.04–3.09)	0.35

Abbreviations: AZA: azathioprine; CI: confidence interval; CNI: calcineurin inhibitors; CPA: cyclophosphamide; F: fixed effect; GC: glucocorticoid; IS: immunosuppressive agents; Largest effect: effect of the largest individual study in the meta-analysis; M: model; No: Number; MMF: Mycophenolate mofetil; OR: odds ratios; R: random effect; RR: relative risk; TAC: tacrolimus.

**Table 10:** Progression to ESRD during treatment of lupus nephritis (Network meta-analysis).

Comparison	Author, Year	Reported Summary Effect (95% CI)
GC vs. CPA + GC	Palmer, et al., 2017 [10]	2.40 (1.05–5.48)
CPA + AZA vs. GC	Singh, et al., 2016 [16]	0.23 (0.07–0.64)
High GC vs. AZA + GC	Singh, et al., 2016 [16]	2.15 (1.06–4.10)
High GC vs. MMF	Singh, et al., 2016 [16]	4.54 (1.45–17.31)
High CPA + GC vs. High GC	Singh, et al., 2016 [16]	0.24 (0.06–0.71)
Plasma exchange vs. CPA	Palmer, et al., 2017 [10]	2.92 (0.31–27.8)

Abbreviations: AZA: azathioprine; CPA: cyclophosphamide; GC: glucocorticoid; MMF: mycophenolate mofetil.

**Table 11:** Infection during induction therapy (compared with Network meta-analysis).

Comparison	Author, Year	M	Type of Metrics	Reported Summary Effect (95% CI)	NMA Author, Year	NMA Reported Summary Effect (95% CI)	Reported *p* Value	I2 (*p* Value)	No. of Significant Study/Total Study	No. of Cases/Controls	Largest Effect (95% CI)	*p* Value (Largest)
MMF + GC vs. CPA + GC	Tunnicliffe, et al., 2018 [5]	R	RR	1.02 (0.67–1.54)	Lee, et al., 2015 [12]	0.87 (0.54–1.53)	0.94	0% (0.50)	0/9	699/351	1.22 (0.68–2.20)	-
TAC + GC vs. CPA + GC	Zhou, et al., 2019 [11]	F	OR	0.77 (0.42–1.43)	Lee, et al., 2015 [12]	0.49 (0.21–1.23)	0.41	-	0/5	-	-	-
CNI + GC vs. CPA + GC	Zhang, et al., 2016 [13]	F	RR	0.65 (0.43–0.98)	Palmer, et al., 2017 [10]	0.57 (0.28–1.16)	0.04	0% (0.53)	0/5	194/97	1.09 (0.32–3.73)	0.9
CPA + GC vs. GC	Tunnicliffe, et al., 2018 [5]	R	RR	0.87 (0.50–1.51)	Palmer, et al., 2017 [10]	0.81 (0.34–1.94) *	0.61	0% (0.59)	0/6	291/113	1.12 (0.53–2.40)	-
Oral CPA vs. IV CPA	Tunnicliffe, et al., 2018 [5]	R	RR	1.16 (0.47–2.90)	Palmer, et al., 2017 [10]	1.12 (0.43–2.94)	0.75	0% (0.35)	0/2	67/34	0.60 (0.11–3.19)	-
Low CPA vs. High CPA	Tian, et al., 2017 [17]	F	RR	0.74 (0.56–0.98)	Bae, et al., 2019 [14]	0.62 (0.29–1.25)	0.03	20% (0.28)	1/7	655/348	0.80 (0.47–1.35)	0.39
TAC + GC vs. MMF + GC	Zhou, et al., 2019 [11]	R	OR	0.95 (0.06–16.03)	Lee, et al., 2015 [12]	0.56 (0.24–1.30)	0.97	-	-	-	-	-

Abbreviations: CI: confidence interval; CNI: calcineurin inhibitors; CPA: cyclophosphamide; F: fixed effect; GC: glucocorticoid; Largest effect: effect of the largest individual study in the meta-analysis; M: model; No: Number; NMA: Network meta-analysis; MMF: Mycophenolate mofetil; OR: odds ratios; R: random effect; RR: relative risk; TAC: tacrolimus. * results of GC vs. CPA + GC.

**Table 12:** Infection during induction and maintenance therapy in lupus nephritis.

Comparison	Author, Year	M	Type of Metrics	Reported Summary Effect (95% CI)	Reported *p* Value	I2 (*p* Value)	No. of Significant Study/Total Study	No. of cases/Controls	Largest Effect (95% CI)	*p* Value (Largest)
Induction therapy										
CPA vs. Others										
MMF + TAC + GC vs. CPA + GC	Tunnicliffe, et al., 2018 [5]	R	RR	0.80 (0.22–2.94)	0.74	57% (0.13)	2/2	402/201	1.80 (1.34–2.42)	<0.001
MMF + GC vs. Oral CPA + GC	Tunnicliffe, et al., 2018 [5]	F	RR	0.38 (0.08–1.79)	0.22	-	0/1	62/30	0.38 (0.08–1.79)	0.22
Long CPA vs. Short CPA	Tunnicliffe, et al., 2018 [5]	R	RR	1.00 (0.07–14.90)	1.0	-	0/1	40/20	1.00 (0.07–14.90)	1.0
AZA + CPA+ GC vs. GC	Tunnicliffe, et al., 2018 [5]	F	RR	0.48 (0.10–2.30)	0.36	-	0/1	29/7	0.48 (0.10–2.30)	0.36
CNI vs. Others										
TAC vs. Others	Karaaij, et al., 2016 [21]	F	RR	0.91 (0.69–1.19)	-	26.9% (0.242)	0/5	693/-	1.14 (0.81–1.60)	-
AZA vs. TAC	Tunnicliffe, et al., 2018 [5]	F	RR	1.26 (0.30–5.22)	0.75	-	0/1	70/34	1.26 (0.30–5.22)	0.75
MMF + GC vs. TAC + GC	Tunnicliffe, et al., 2018 [5]	R	RR	2.14 (0.93–4.92)	0.60	0% (0.28)	0/2	190/94	1.70 (0.52–5.58)	0.53
Biologic agents										
Rituximab + CPA vs. Rituximab	Tunnicliffe, et al., 2018 [5]	F	RR	0.09 (0.07–12.38)	0.94	-	0/1	19/9	0.09 (0.07–12.38)	0.94
Rituximab + MMF vs. MMF	Tunnicliffe, et al., 2018 [5]	F	RR	1.00 (0.48–2.08)	1.0	-	0/1	144/72	1.0 (0.48–2.08)	1.0
Rituximab + IS vs. IS	Shamliyan, et al., 2017 [29]	F	RD	0.03 (−0.03–0.08)	-	0% (0.78)	0/1	-	0.03 (−0.03–0.08)	-
Abatacept + IS vs. placebo + IS	Tunnicliffe, et al., 2018 [5]	R	RR	1.29 (0.81–2.04)	0.28	0% (0.62)	0/2	432/168	1.22 (0.73–2.03)	-
Orcrelizumab + IS vs. placebo + IS	Tunnicliffe, et al., 2018 [5]	F	RR	1.14 (0.95–1.36)	0.17	-	0/1	378/125	1.14 (0.95–1.36)	0.17
Sirukumab + IS vs. placebo + IS	Tunnicliffe, et al., 2018 [5]	F	RR	0.93 (0.66–1.32)	0.70	-	0/1	25/4	0.93 (0.66–1.32)	0.70
Belimumab vs. placebo	Kandala, et al., 2013 [27]	R	RR	0.85 (0.57–1.28)	-	-	0/3	2133/675	-	-
Others										
MMF + CPA + GC vs. CPA + GC	Tunnicliffe, et al., 2018 [5]	F	RR	0.37 (0.14–0.93)	0.035	-	1/1	82/40	0.37 (0.14–0.93)	0.035
Plasma exchange + IS vs. IS	Tunnicliffe, et al., 2018 [5]	R	RR	0.69 (0.35–1.37)	0.29	0% (0.36)	0/2	125/65	0.65 (0.32–1.30)	-
Plasma exchange vs. IS	Tunnicliffe, et al., 2018 [5]	F	RR	0.40 (0.02–8.78)	0.56	-	0/1	20/11	0.40 (0.02–8.78)	-
GC vs. reduced GC	Tunnicliffe, et al., 2018 [5]	F	RR	4.64 (0.57–38.00)	0.15	-	0/1	81/39	4.64 (0.57–38.00)	0.15
MMF vs. AZA	Deng, et al., 2019 [18]	R	RR	0.61 (0.29–1.30)	0.20	84% (0.0004)	1/4	255/124	1.26 (0.88–1.79)	-
AZA + GC vs. GC	Tunnicliffe, et al., 2018 [5]	R	RR	3.56 (0.46–27.79)	0.23	0% (0.59)	0/2	42/16	2.00 (0.11–37.22)	-
Maintenance therapy										
AZA vs. MMF	Tunnicliffe, et al., 2018 [5]	F	RR	1.08 (0.60–1.96)	0.79	0% (0.87)	0/3	412/209	1.22 (0.57–2.62)	-
CNI vs. AZA	Zhang, et al., 2016 [13]	F	RR	0.56 (0.28–1.10)	0.09	0% (0.34)	0/2	139/69	1.06 (0.23–4.89)	>0.99
AZA vs. GC	Tunnicliffe, et al., 2018 [5]	F	RR	2.18 (1.01–4.73)	0.048	-	1/1	69/36	2.18 (1.01–4.73)	0.048
GC withdrawal vs. GC continuous	Tunnicliffe, et al., 2018 [5]	R	RR	0.57 (0.06–5.03)	0.61	-	0/1	15/8	0.57 (0.06–5.03)	0.61

Abbreviations: AZA: azathioprine; CI: confidence interval; CNI: calcineurin inhibitors; CPA: cyclophosphamide; F: fixed effect; GC: glucocorticoid; IS: immunosuppressive agents; Largest effect: effect of the largest individual study in the meta-analysis; M: model; No: Number; MMF: Mycophenolate mofetil; OR: odds ratios; R: random effect; RR: relative risk; TAC: tacrolimus.

**Table 13:** Infection during treatment of lupus nephritis (Network meta-analysis).

Comparison	Author, Year	Reported Summary Effect (95% CI)
MMF + GC vs. CPA + GC	Palmer, et al., 2017 [10]	1.23 (0.84–1.77)
MMF + CNI + GC vs. CPA + GC	Palmer, et al., 2017 [19]	3.59 (0.66–19.5)
Low CPA + GC vs. MMF + GC	Bae SC, et al., 2019 [14]	0.73 (0.29–1.66)
MMF + GC vs. High CPA + GC	Bae SC, et al., 2019 [14]	0.85 (0.52–1.39)
AZA + GC vs. CPA + GC	Palmer, et al., 2017 [10]	0.34 (0.10–1.13)
MMF + GC vs. GC	Tang, et al., 2018 [25]	5.50 (1.00–30.1)
CNI + GC vs. GC	Tang, et al., 2018 [25]	5.0 (1.1–22.6)

Abbreviations: AZA: azathioprine; CNI: calcineurin inhibitors; CPA: cyclophosphamide; GC: glucocorticoid; MMF: mycophenolate mofetil.

**Table 14:** Relapse during maintenance therapy (compared with Network meta-analysis).

Comparison	Author, Year	M	Type of Metrics	Reported Summary Effect (95% CI)	NMA Author, Year	NMA Reported Summary Effect (95% CI)	Reported *p* Value	I2 (*p* Value)	No. of Significant Study/Total Study	No. of cases/Controls	Largest Effect (95% CI)	*p* Value (Largest)
MMF vs. AZA	Deng, et al., 2019 [18]	F	RR	0.75 (0.54–1.05)	Singh, et al., 2016 [4]	0.59 (0.38–0.90)	0.09	0% (0.72)	1/7	558/270	0.55 (0.31–0.99)	0.03
CNI vs. AZA	Zhang, et al., 2016 [13]	F	RR	0.44 (0.10–1.89)	Palmer, et al., 2017 [10]	0.64 (0.22–1.88)	0.27	0% (0.54)	0/2	139/69	0.64 (0.22–1.88)	0.49
AZA vs. CPA	Tunnicliffe, et al., 2018 [5]	R	RR	0.79 (0.34–1.85)	Palmer, et al., 2017 [10]	1.68 (0.51–5.51) *	0.59	-	0/1	39/20	0.79 (0.34–1.85)	0.59

Abbreviations: AZA: azathioprine; CI: confidence interval; CNI: calcineurin inhibitors; CPA: cyclophosphamide; F: fixed effect; Largest effect: effect of the largest individual study in the meta-analysis; M: model; No: Number; NMA: Network meta-analysis; MMF: Mycophenolate mofetil; R: random effect; RR: relative risk. * results of CPA vs. AZA.

**Table 15:** Mortality during induction and maintenance therapy of lupus nephritis (Network meta-analysis).

Comparison	Author, Year	Reported Summary Effect (95% CI)
MMF + CNI + GC vs. CPA + GC	Palmer, et al., 2017 [10]	1.00 (0.02–52.8)
Plasma exchange + GC vs. CPA +GC	Palmer, et al., 2017 [10]	8.21 (0.22–3.04)

Abbreviations: CNI: calcineurin inhibitors; CPA: cyclophosphamide; GC: glucocorticoid; MMF: mycophenolate mofetil.

**Table 16:** Malignancy during induction and maintenance therapy of lupus nephritis.

Comparison	Author, Year	M	Type of Metrics	Reported Summary Effect (95% CI)	Reported *p* Value	I2 (*p* Value)	No. of Significant Study/Total Study	No. of Cases/Controls	Largest Effect (95% CI)	*p* Value (Largest)
Induction therapy										
MMF + GC vs. CPA + GC	Tunnicliffe, et al., 2018 [5]	R	RR	0.65 (0.11–3.86)	0.64	-	0/1	364/180	0.65 (0.11–3.86)	0.64
CPA + GC vs. GC	Tunnicliffe, et al., 2018 [5]	R	RR	0.82 (0.07–9.90)	0.87	26% (0.24)	0/2	147/39	2.69 (0.15–49.06)	-
CNI + GC vs. CPA + GC	Tunnicliffe, et al., 2018 [5]	F	RR	5.00 (0.26–97.70)	0.29	-	0/1	38/19	5.00 (0.26–97.70)	-
Low vs. High CPA	Tunnicliffe, et al., 2018 [5]	R	RR	1.44 (0.09–23.31)	0.80	41% (0.19)	0/2	206/88	5.47 (0.30–99.28)	-
Long vs. Short CPA	Tunnicliffe, et al., 2018 [5]	R	RR	3.00 (0.13–69.52)	0.49	-	0/1	40/20	3.00 (0.13–69.52)	0.49
Belimumab * vs. placebo	Borba, et al., 2014 [30]	R	RR	1.50 (0.25–9.17)	0.66	0% (0.71)	0/3	1349/674	2.01 (0.18–22.09)	-
Maintenance therapy										
AZA vs. MMF	Tunnicliffe, et al., 2018 [5]	F	RR	4.04 (0.45–36.07)	0.21	0% (0.82)	0/3	370/188	3.11 (0.13–75.47)	-

Abbreviations: AZA: azathioprine; CI: confidence interval; CNI: calcineurin inhibitors; CPA: cyclophosphamide; F: fixed effect; GC: glucocorticoid; Largest effect: effect of the largest individual study in the meta-analysis; M: model; No: Number; MMF: Mycophenolate mofetil; OR: odds ratios; R: random effect; RR: relative risk. * Belimumab 10 mg/kg.

**Table 17:** Malignancy during treatment of lupus nephritis (Network meta-analysis).

Comparison	Author, Year	Reported Summary Effect (95% CI)
AZA + GC vs. CPA + GC	Palmer, et al., 2017 [10]	4.67 (0.61–37.7)
Oral CPA + GC vs. IV CPA + GC	Palmer, et al., 2017 [10]	7.77 (0.81–74.9)

Abbreviations: AZA: azathioprine; CPA: cyclophosphamide; GC: glucocorticoid; IV: intravenous.

## Data Availability

Data are available from the corresponding author on a reasonable request.
